# Forest productivity in southwestern Europe is controlled by coupled North Atlantic and Atlantic Multidecadal Oscillations

**DOI:** 10.1038/s41467-017-02319-0

**Published:** 2017-12-20

**Authors:** Jaime Madrigal-González, Juan A. Ballesteros-Cánovas, Asier Herrero, Paloma Ruiz-Benito, Markus Stoffel, Manuel E. Lucas-Borja, Enrique Andivia, Cesar Sancho-García, Miguel A. Zavala

**Affiliations:** 1 Forest Ecology and Restoration Group, Department of Life Sciences, Universidad de Alcalá, 28805 Alcalá de Henares, Madrid Spain; 2 Climate Change Impacts and Risks in the Anthropocene (C-CIA), Institute for Environmental Sciences, University of Geneva, Boulevard Carl-Vogt 66, 1205 Geneva, Switzerland; 3 Department of Earth Sciences, Dendrolab, University of Geneva, Rue des Maraîchers 13, 1205 Geneva, Switzerland; 4 School of Forestry, Northern Arizona University, Flagstaff, AZ 86011 USA; 5 Department of Plant Biology and Ecology, Faculty of Science and Technology, University of Basque Country, 48940 Leioa, Basque Country Spain; 6 Department F.-A. Forel for Environmental and Aquatic Sciences, University of Geneva, Boulevard Carl-Vogt 66, 1205 Geneva, Switzerland; 7 Department of Agricultural Technology and Science and Genetics, ETSIAM, Universidad de Castilla-La Mancha, Campus Universitario s/n, 02071 Albacete, Spain; 8 Department of Applied Mathematics, University of Valladolid, Avenida del Valle Esgueva 6, 47011 Valladolid, Spain

## Abstract

The North Atlantic Oscillation (NAO) depicts annual and decadal oscillatory modes of variability responsible for dry spells over the European continent. The NAO therefore holds a great potential to evaluate the role, as carbon sinks, of water-limited forests under climate change. However, uncertainties related to inconsistent responses of long-term forest productivity to NAO have so far hampered firm conclusions on its impacts. We hypothesize that, in part, such inconsistencies might have their origin in periodical sea surface temperature anomalies in the Atlantic Ocean (i.e., Atlantic Multidecadal Oscillation, AMO). Here we show strong empirical evidence in support of this hypothesis using 120 years of periodical inventory data from Iberian pine forests. Our results point to AMO^+^ NAO^+^ and AMO^−^NAO^−^ phases as being critical for forest productivity, likely due to decreased winter water balance and abnormally low winter temperatures, respectively. Our findings could be essential for the evaluation of ecosystem functioning vulnerabilities associated with increased climatic anomalies under unprecedented warming conditions in the Mediterranean.

## Abstract (web summary)

The North Atlantic Oscillation (NAO) drives biological responses in terrestrial ecosystems through oscillatory modes of climatic variability. Here, the authors show how landscape scale productivity responses to NAO are contingent upon the Atlantic Multidecadal Oscillation in southwestern Europe.

## Introduction

Forest productivity represents a major component of the forest carbon sink, which has been estimated to be as much as 30% of anthropogenic carbon emissions (2 Pg C per year) over the past few decades^1^. Water-limited forests in the Mediterranean Basin are not an exception in this regard, and have indeed been shown to contribute moderately to carbon sequestration, despite the existence of intrinsic local environmental variability^2^. Recent findings, however, suggest that increased intensity and frequency of drought anomalies might expose water-limited forests to unprecedented vulnerabilities, which in turn might jeopardize or even reverse their global role as carbon sinks^3,4^. Developing adaptive strategies to preserve the carbon sink role of water-limited forests in the long term requires an in-depth understanding of how forest productivity responds to drought variability^5^.

Over the last decades, research on the global climate system has bridged global atmospheric phenomena to oscillatory modes of climatic variability^6^. The North Atlantic Oscillation (NAO) is one such phenomenon directly related to drought anomalies in North America and Europe^7–13^. The NAO emerges from the dipole of atmospheric masses at the sea surface level between the Azores and Iceland acting as a latitudinal switch of oceanic influences^14^. A weakened dipole (i.e., negative NAO) allows moist westerly winds to reach southwestern European latitudes, and thus, facilitates the arrival of abundant rainfall and moderate temperatures. By contrast, the strengthening of the dipole (e.g., a positive NAO phase) favors the occurrence of anticyclonic weather conditions in the region as well as increased frequency of drought anomalies, nocturnal frosts, and extreme daytime heat^15^. Two contrasting response patterns to NAO have been reported from north to south in Western Europe;—positive responses in northern boreal/temperate forests^16^ and negative ones in the western Mediterranean Basin^17^. In southern European forests, ecological responses to NAO have been related to plant phenology^18^, fruit production^19^, seed masting^20^, tree growth^2,17,21–23^, and pest occurrence^24^. All these processes are directly or indirectly associated with soil water recharges during winter, needed to support forest functions when available energy becomes large enough to maintain carboxylation and xylogenesis^21^. The signature of NAO phases on tree growth, however, has been shown to vary over longer timescales^25^, and this, has given rise to the formulation of new hypotheses where NAO teleconnections (i.e., linkages between distant climatic anomalies due to North Atlantic sea surface level pressure changes) are coupled to other atmospheric/oceanic influences and/or modes^26,27^. Understanding teleconnections associated with coupling of NAO to other climate modes of variability thus represent a promising research line toward more accurate assessments of the carbon sink role of forests in the long term.

The Atlantic Multidecadal Oscillation (AMO) might be such a major climate mode associated with periodical anomalies of sea surface temperatures (SSTs) in northern, extratropical latitudes. In North America, Europe, and the Sahel region, relationships between the AMO and seasonal weather anomalies have been described^28–30^, as has the subsequent signature in long-living organisms such as corals^31^ or trees^32^. AMO^+^ phases (i.e., positive anomalies of SST) have been linked to heat waves and squall formation over the European continent^33^. More recently, the AMO has also been related to non-stationary patterns of precipitation and temperature in both North America and Western Europe^28,34^. Thus, it might be hypothesized that forest productivity in southwestern Europe is controlled by anomalies in the winter water balance emerging from coupled patterns of NAO and AMO. The AMO could be acting as a SST-based forcing of the oceanic moisture advection, whereas the NAO acts as the latitudinal switch for the income of such oceanic influences over the continent. Moreover, AMO and NAO are known to affect the probability of cold and heat extremes, which can also affect tree growth via other teleconnections. For instance, during phases with abnormally cold SSTs (i.e., AMO^−^ phase), the induced cooling might instead exceed the predominant role of water thus reversing forest growth responses to the NAO. Surprisingly, empirical evidence of such influence induced by a coupling of NAO and AMO is lacking, probably because most of the available biological records barely cover a full AMO cycle, which is in the order of 70–80 years.

Here we demonstrate that the coupling of NAO and AMO substantially influences long-term volume increment (VI), a surrogate of forest productivity, at the landscape scale in southwestern Europe. The study is based on detailed historical forest inventory data consisting of periodic wood volume measurements since the end of the 19th century in a number of forest spatial units (hereafter forest units) comprising hundreds of hectares and thousands of trees within five representative forest landscapes of the Iberian Peninsula. The five forest landscapes considered cover a total extension of >33,000 ha and include three different ecosystems dominated by contrasted and widespread pine species in Europe (*Pinus sylvestris* *L.*, *P. pinaster* *Aiton*, and *P. nigra* *Arn.*). First, VI was regressed against the interactive effects of the NAO and AMO, and accounts for thinning (Th), tree density (TD), mean tree size (MTS), and calendar year (as a predictor variable for productivity responses linked to global changes^35^). Second, structural equation modeling (SEM^36^) allowed us to test the hypothesis that, over the last 120 years, the AMO coupled to the NAO has affected VI indirectly through a direct influence on winter water balance (i.e., precipitation-potential evapotranspiration, P-PET), which is critical for soil water recharge in Mediterranean ecosystems; thus tree growth and ecosystem functioning are sensitive to fluctuations in the balance between precipitation and evapotranspiration during winter^21^. Finally, empirical support to a direct relationship between NAO and AMO coupling and winter water balance is provided using available local climate data from representative nearby meteorological stations.

## Results

### North Atlantic Oscillations as forest productivity drivers

**Fig. 1:**
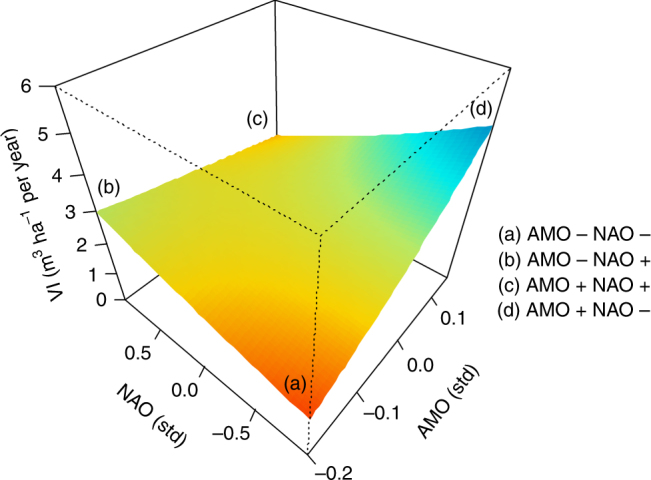
Volume increment as function of NAO and AMO. Three-dimensional regression plot showing predicted wood volume increment (VI, as surrogate of forest productivity) responses to standardized North Atlantic Oscillation (NAO) and Atlantic Multidecadal Oscillation (AMO) interactive effects. The colored layout (i.e., from red to blue increasing VI values) represents the marginal response curve when the other variables in the best model are kept to mean values (i.e., mean tree size and the calendar year). Letters indicate the areas in the response curve related to each potential sign of NAO and AMO combination

A first mixed-effects model was designed to unravel the simultaneous effects of North Atlantic climate modes, last-century global changes, and forest factors on VI. Model selection using the Akaike Information Criterion (AIC) supported a negative effect of MTS (see Supplementary Table 1 and sign of parameter estimate in Supplementary Table 2). A positive last-century linear trend of VI was also supported, whereas TD and species did not receive enough support to be included in the final model, indicating a limited effect of both variables on VI (Supplementary Table 1 and Supplementary Table 2). Finally, model selection strongly supported the interactive effect of NAO and AMO driving VI (Supplementary Table 1). The supported model showed high goodness of fit as shown by both the observed vs. predicted plot (Supplementary Fig. 1, and the high conditional *R* ^2^ (i.e., 0.78)). The most negative effect of NAO and AMO coupling was especially noticeable during NAO^−^AMO^−^ phases. For these periods, the model points to reductions in as much as 60% of mean VI, provided that the other predictor variables are kept at average values (Fig. 1a). Slighter reductions of up to 30% of mean VI were also observed during AMO^+^ and NAO^+^ phases (Fig. 1c). By contrast, whereas VI almost doubled during AMO^+^NAO^−^ phases (Fig. 1d), the model only suggests moderate impacts of AMO^−^NAO^+^ phases on VI (Fig. 1b).

### Long-term mechanistic evidence via winter water balance

**Fig. 2:**
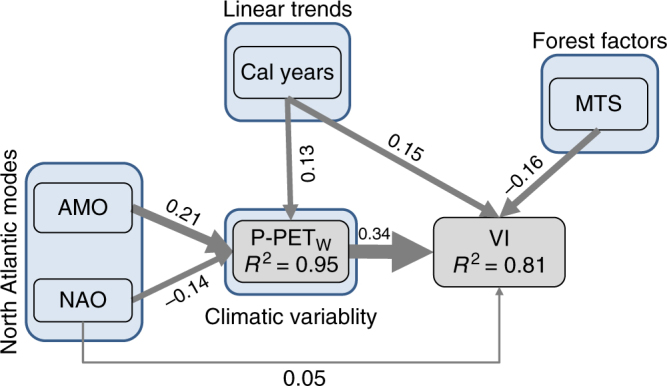
Mechanistic model for climate-productivity relationships. Results of the Structural Equation Models (SEMs) exploring the effects of North Atlantic modes (NAO and AMO) and last-century linear trends (calendar year) on the winter water balance (P-PET), and the effects of mean tree size (MTS) and local climatic variability (winter water balance, P-PET) on wood volume increments (VI, as surrogate of forest productivity). An unaccounted link between NAO and VI was included after evaluation of potential missing paths in the initial hypothesis. Boxes represent measured variables and arrows unidirectional relationships among variables. Only significant paths (*P* ≥ 0.05) among all possible paths are shown. The thickness of the significant paths has been scaled based on the magnitude of the standardized regression coefficient (showed beside arrows). *R* ^2^ conditional for the forest productivity model (based on the variance of both the fixed and random effects) is given within the P-PET and the VI box

To better understand whether the apparent correlation between NAO/AMO variability and forest productivity is mechanistically plausible, we evaluated the hypothesis that coupled NAO and AMO indirectly affect VI through their influence on climatic variability using SEM. We thus considered two separate models. First, VI as a function of winter P-PET, calendar year as well as the forest factor with significant influence in the previous growth model (i.e., MTS). Second, winter P-PET as function of NAO, AMO, and the calendar year. After inclusion of a missing direct path between NAO and VI (standardized coefficient = 0.15) in the initial hypothesis (Fig. 2), the entire structured model resulted in an adequate goodness of fit based on Fisher’s C statistic (*C*_(df__ = 6)_ = 5.31, *P = *0.257). Our results show how NAO and AMO indirectly affect VI through the modification of winter P-PET (standardized coefficients −0.14 and 0.21, respectively), which in turn had a notable direct effect on VI (standardized coefficient = 0.34, Fig. 2). Our analysis also shows a direct positive global change trend in VI (standardized coefficient = 0.15) and a positive indirect influence of global change through the modification of P-PET (standardized coefficient = 0.13).

### North Atlantic modes as drivers of local climatic records

**Fig. 3:**
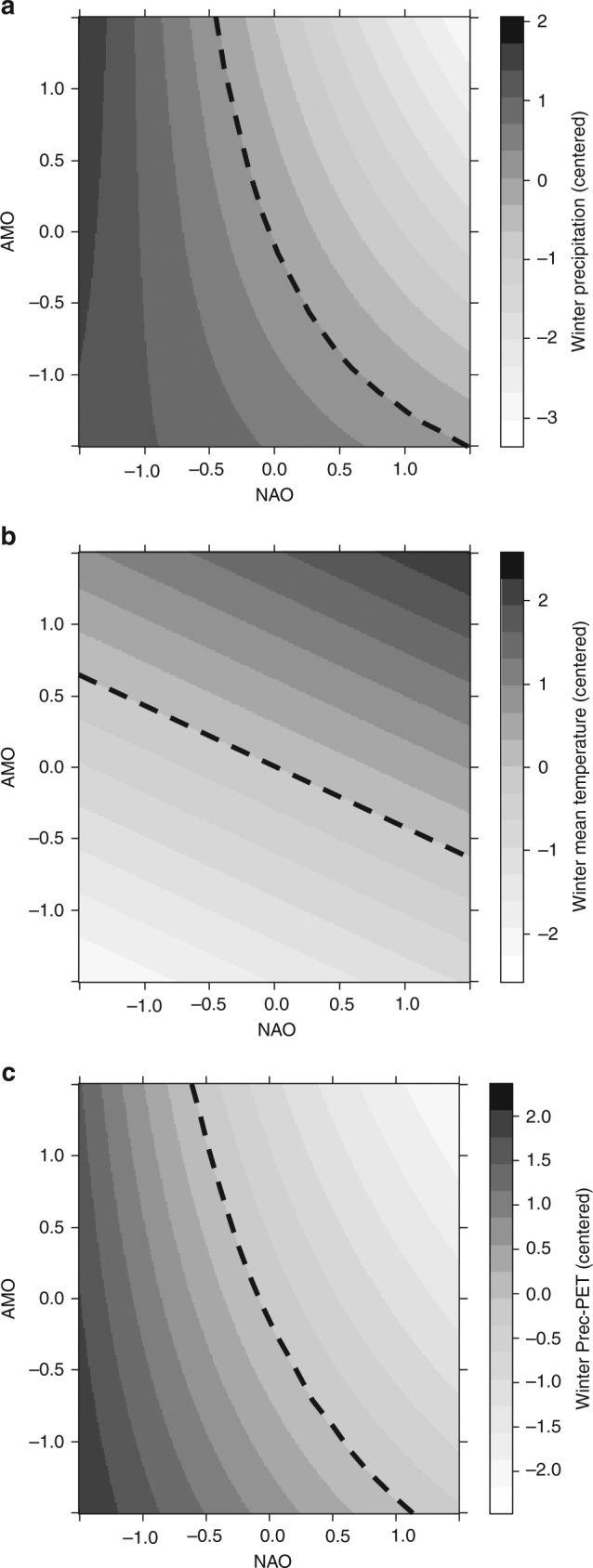
North Atlantic Oscillations as drivers of local climatic variability. Predicted (**a**) winter total precipitation, (**b**) winter mean temperature, and (**c**) winter precipitation (Prec)-potential evapotranspiration (PET) as function of the North Atlantic and the Atlantic Multidecadal oscillations (NAO and AMO, respectively) using generalized linear models (gamma distribution of error and inverse link) in cases **a** and **b**, and a linear model in case **c**. Dependent variables were both centered (*y*−*y*_mean_) for an easier interpretation of colored isoline plots (dotted line represents *y*_mean_)

The interactive effects between the NAO and AMO on local climate were analyzed separately at annual resolution (see Supplementary Table 3 for further information of meteorological stations considered). The supported models were all coherent with previous findings (Fig. 1) on the relationship between VI and long-term climate at decadal timescales (Fig. 2). Our results supported a significant effect of NAO × AMO on winter precipitation on the one hand (Fig. 3a), and winter P-PET on the other hand (Fig. 3c). In both cases, winter water availability increased toward NAO^−^ (see parameter estimates in Supplementary Table 4) and, on the contrary, minimum winter water balance during coupled NAO^+^ and AMO^+^ phases. During NAO^−^ and AMO^−^ phases, our results point to the highest winter water balance, mostly due to a strong significant decrease in winter temperatures (Fig. 3b), and thus to reduced potential evapotranspiration.

## Discussion

Last-century VI in Iberian pinelands strongly resembles coupled patterns of NAO and AMO phases and thus confirms that the impact of a NAO signature on VI is subsidiary of the low-frequency SST anomalies described by the AMO index. Moreover, our results point to significant relationships between North Atlantic climate modes coupling and local climate, and thus strongly support the hypothesized control of NAO and AMO on VI through climate. Large-scale simulations of net primary productivity and remote sensing evidence have recently provided support to this hypothesis^27^, albeit with a reduced time scope that precluded the detection of important low-frequency modes of climate variability such as the AMO. Our results, by contrast, are supported by a number of decadal field measurements over the 20th century in Iberian forests. As the data set comprises comprehensive historical information such as TD, MTS, Th, and global change trends, we add strong evidence to the initial hypothesis postulating the existence of important teleconnections and a coupling of climate modes over the North Atlantic. Indeed, we demonstrate that this coupling becomes apparent in the form of rank reversals of VI responses to NAO following changes in AMO: i.e., the well-known negative response of tree growth to NAO is manifested only during neutral-positive SST anomalies identified with AMO^+^ phases. On the contrary, a slightly positive response to NAO can emerge during the AMO antiphase (AMO^−^), when SSTs are below the long-term mean. These findings will have profound implications on our understanding of forest carbon fluxes, and for the development of accurate estimations of forest carbon sinks and vulnerability to increasing aridity.

The control exerted by NAO and AMO coupled patterns on VI is driven by anomalies in the winter water balance. The SST increase in subtropical areas during AMO^+^ phases results in a greater moisture-holding capacity of the atmosphere^37,38^, with a likely influence of the Atlantic SST on circulation regimes in the extra-tropics and the occurrence of extreme drought conditions^39^, as well as other drought-induced weather anomalies^28^ over Europe, namely anticyclonic and blocking conditions^38^. In addition, the location of high-low pressure systems over the North Atlantic Ocean (i.e., NAO index) also exerts major control on the storm paths and on winter weather regimes in Europe^40–43^. In this context, AMO^+^ and NAO^+^ phases match with notable reductions in VI, which in turn are linked to extremely negative winter water balances (reflected in extremely dry conditions during the 1940s and 1990s on the Iberian Peninsula^44^). By contrast, the combination of AMO^+^ and NAO^−^ phases obviously coincides with abundant winter precipitation (as observed during the late 1930s and in the 1950s^45–48^) and thereby lead to increases in VI. Analyses on seasonality trends clearly point to a significant relationship between NAO^−^-like modes and the frequency of wet and dry conditions^48^, and are thus in agreement with the concept of the interactive effects of NAO and AMO^−^-like modes presented here on weather regimes over the Iberian Peninsula.

The coupling of NAO and AMO indeed is crucial as it not only integrates water constraints, but also temperature limitations on VI. The combination of AMO^−^ and NAO^+^ phases corresponds with slight increments in VI and a moderate winter water balance, whereas AMO^−^and NAO^−^ phases match with the strongest reductions in VI and paradoxically the highest winter water balance. This strong decrease in VI, coupled to the wettest conditions and low temperatures, suggests increased energy limitations in agreement with temperature anomalies during AMO^−34^ and NAO^−49^ phases. Temperature is an important driver of tree growth in elevated inland regions of southern Europe^50,51^, as low temperatures reduce photosynthetic activity through limitations on RUBISCO, carboxylation, and xylogenesis^52^. Thus, low temperatures will strongly limit tree growth in pine species in southwestern Europe as recently shown for Iberian populations of *P. sylvestris* ^53^. Thus, slight increments in temperature motivated by anticyclonic conditions associated to NAO^+^ might determine, as hypothesized, positive responses in forest productivity during AMO^−^ phases.

It is important to note that, although winter water balance is decisive for the recharge of available water for plants in Mediterranean biomes^54^, climatic variability during the remainder of the year may also play a role in driving forest productivity. For example, temperature and precipitation during early spring have been successfully correlated to tree growth in the studied and similar forests^55^. Thus, a further broadening of the scope of climatic indices or even an inclusion of other, new climatic phenomena might enhance our understanding of forest-climate relationships further. This will, however, be an arduous task since climatic patterns remain, in general, only poorly understood, and since even less is known about their teleconnections with local climate on the continents. The connection between global modes and local climate as well as their effects on forest performance will likely become a major research topic at the interface between climatology and ecology in the future. It will improve our ability to evaluate accurately potential climatic impacts on forest functions and services, especially in vulnerable areas such as the western Mediterranean Basin.

Our findings imply that a large proportion of forest productivity at the regional or potentially even at the hemispheric scales could be explained by large-scale, multidecadal internal climate-ocean variability. We provide strong physical evidence of the impact of the coupled low-frequency, ocean-atmosphere variability mode described herein on southern Europe pine forest functioning in terms of VI. These results have direct implications for the assessment of terrestrial carbon sink vulnerability under climate change. The combination NAO^+^ and AMO^+^ have been shown to result in reduced winter water balance, which added to the growing vapor pressure deficit associated with enhanced atmospheric water demands due to climate warming, may exacerbate drought impacts on forest ecosystems. Besides reductions in VI, global change-type drought (or amplified drought by global warming) can result in increased tree mortality and reduced regeneration, thereby limiting the ability of forest ecosystems to act as carbon sinks and favoring vegetation shifts from forests to treeless communities^56^. Furthermore, a more persistent NAO^+^ phase under climate change^57^ could aggravate the deleterious effects of the interaction between the NAO and the AMO, particularly in multidecadal periods of prevailing AMO^+^, through longer, and more intense, dry periods. Up until now, however, calendar year trends had a direct and indirect positive effect on VI. On the one hand, a slight positive trend in winter P-PET has presumably contributed to enhance forest productivity until the beginning of the 21st century. On the other hand, a positive last-century trend in forest productivity reveals the existence of other factors likely associated to global change trends such as increments in temperature, changes in precipitation patterns, growing atmospheric CO_2_ concentration^58^, and/or nitrogen depositions^59,60^. Although a linking of calendar year to global change trends is commonly done in literature^35^, further research is needed to clarify actual variables and mechanisms, climatic and non-climatic, underpinning forest productivity trends in the long term, since factors like age-related trends in radial growth or soil moisture due to cumulative effects of water balance (P-PET + runoff)) could affect forest productivity trends as well.

Understanding the underlying causes of net primary productivity responses to climatic variability is critical to predict and establish realistic long-term mitigation targets for each region as well as to implement flexible adaptation and management measures^61^. Thinning is one of the most effective and applied adaptation measures in water-limited forests, as it promotes forest resilience to drought impacts^62,63^. The oscillatory nature of the NAO and the AMO can allow detection of periods with high drought risk and thus allow for the development of anticipatory adaptation measures such as selective Th, adjustment of regeneration, cutting age cycles, and stand species diversification. Anticipatory measures are key for optimizing management effectiveness and efficiency as the positive effects of Th decrease with increasing time since the last application^62,63^. Advancement of drought risk periods can thus enhance forest resilience^64^ by allowing us to adjust forest structure and regeneration periods to more realistic and flexible climate scenarios. The achievement of forest resilience is particularly critical given the high risk of irreversible ecosystem degradation and aridification under climate warming in this region.

## Methods

### Forest management projects

The study was conducted using historical forest inventory data recorded in five extensive pine forest landscapes in central-southern Spain (i.e., *c*. 33,000 hectares). Location and primary environmental data can be found in Supplementary Table 5 and Supplementary Fig. 2. The five forest landscapes considered in this study exhibit public ownership and have similar management prescriptions following shelterwoods with progressive Th to facilitate tree regeneration by keeping a protective adult tree cover. Prior to the application of management practices, the foresters divided forest landscapes into a number of forest units (53 forest units distributed in the five forest landscapes, see Supplementary Table 5) that can be recognized as pseudoreplicates of a whole forest landscape because each one includes a representative chronosequence of the rotation period. The area of forest units, with an average value of ~550 ha, depends on the size of the particular forest landscape.

The forest landscapes studied are representative of the most abundant pine tree species in southwestern Europe (Supplementary Table 5): *P. pinaster*, *P. sylvestris*, and *P. nigra*. *P. pinaster* is an abundant well-adapted tree species to water scarcity, whereas the other species, *P. sylvestris* and *P. nigra*, are mountain pine species in the Mediterranean with Eurosiberian and supra-Mediterranean distribution ranges, respectively^65^. The different species tend to occupy different lithologies: while *P. pinaster* and *P. sylvestris* grow in acidic soils on sandy dunes and granite lithologies, respectively, *P. nigra* grow in neutral-basic soils in limestone. In all cases, soils are generally poorly developed, with thin organic horizons and low nutrient contents. Furthermore, *P. sylvestris* is distributed in mountain systems covering an altitudinal range of 1000–1900 m.a.s.l., and, therefore slopes and climatic conditions can vary locally. In the case of *P. pinaster* and *P. nigra*, forests generally occupy flat areas where local environmental variation is lower. In general, these forests are monospecific, yet other tree and shrub species appear to occupy a secondary role in marginal areas and ecotones.

### Biotic information

Volume increment, as a surrogate of forest productivity, was computed using available information on decadal wood volume stocks and management practices recorded periodically since the turn of the 19th century (or the beginning of the 20th century) in the studied forest landscapes. Wood volume stocks were assessed as the sum of every individual tree wood volume in each forest unit. It was therefore necessary to estimate first individual tree wood volume through the most basic trunk dimensions: i.e., diameter at breast height (i.e., DBH) and height. In these management plans, a set of trees that were representative of different diameter classes were periodically (approximately every decade) collected and measured to accurately set the relationship between DBH and height and tree wood volume. After felling, tree bark was removed and the DBH and height were measured. With this simple information, volume can be assessed by applying a cylinder equation, which includes diameter and height as the only parameters. Nonetheless, tree trunks are not perfect cylinders given the progressive reduction of diameter upwards. To correct this deviation from the perfect cylinder, tree trunks were then divided into smaller pieces of 1.5–2 m long. Volumes of each piece were then closer to resembling a cylindrical shape so applying a cylinder equation might then be a good method to estimate volume. Then, volumes of independent trunk pieces were summed up and compared to the first volume estimation obtained from the single tree trunk. The ratio between summed volume assessments using pieces vs. volume on a single trunk was the taper coefficient and thereafter was applied to correct every simple measure of tree volume calculated as a unique cylinder using DBH and height. Taper coefficients were derived to four different diameter classes, which directly contribute to an improved assessment of volume stocks at the forest unit level. It is important to note that DBH and height of every individual tree >20 cm DBH were conveniently measured across all the forest units in every inventory in the five forest landscapes considered. In other words, volume stocks at the forest unit level were assessed as a summation of volumes of all the individual trees. This exhaustive work was possible thanks to the coordination of forest agents of the regional councils and a number of workers who were periodically hired in the nearby localities. Therefore, volume assessments at the unit level represent genuine and highly accurate estimations of volume stocks sampled approximately every decade and covering more than a century. The high quality of this information is a direct legacy of the necessity to accurately arrange forest products and services as pervasive economic resources in these populations since the turn of the 19th century. Nowadays, this information allows us to address important research questions in the fields of ecology and forest science^66–68^.

We used forest-unit-level VIs as a surrogate of replicated forest aboveground productivity comparing wood stocks of consecutive inventories and discounting volume of collected trees, as follows:

1 $${\mathrm{VI}}\,{\mathrm{ = }}\,{{V}}_{\mathrm{2}}\,{\mathrm{-}}\,{{V}}_{\mathrm{1}}\,{\mathrm{ + }}\,{{V}}_{{\mathrm{Th}}},$$

where *V*_2_ is volume in the later inventory, *V*_1_ is volume in the previous inventory, and *V*_Th_ is Th. VI was divided by the area of the corresponding forest unit and then was converted into an annual rate dividing VI per hectare by the number of years between consecutive inventories. This process was necessary since not all of the forest units included in this study have a similar area, and time between consecutive inventories can slightly depart from decadal periods.

We also used other biotic information available in the historical archives as predictor variables of VI: TD (number of trees per hectare) as a simple competition index, MTS (average wood volume of individual trees calculated dividing total wood volume by the number of trees in a forest unit) as a maturity index, and Th as the wood volume collected between consecutive inventories. For both TD and MTS, we considered the initial year of every decadal period. In summary, VI in the studied forests ranged from 0 to 15 m^3^ ha^−1^ per year over the past 120 years (Supplementary Fig. 3b) with maximum rates reaching 15 m^3^ ha^−1^ per year at the mildest site of the Spanish central range (i.e., the Valsaín forest, dominated by *P. sylvestris*). Mean tree size is 0.51 m^3^ (±0.28 m^3^), mean TD 187.5 trees per ha (±111.01 trees per ha), and mean collected wood volume 1.42 m^3^ ha^−1^ per year (±1.42 m^3^ ha^−1^ per year; see last-century patterns in Supplementary Fig. 4).

### Climate information

The smoothed Atlantic Multidecadal Oscillation (AMO) index was retrieved from https://www.esrl.noaa.gov/psd/data/correlation/amon.sm.long.data and it was calculated based on the area weighted average SST from Kaplan SST V2 over the N Atlantic, from 0 to 70°N. The Normalized North Atlantic Oscillation (NAO) index defined as the difference of pressure between Iceland and Gibraltar has been retrieved from https://crudata.uea.ac.uk/cru/data/nao/nao.dat. We used monthly NAO and AMO indices and calculated winter NAO and AMO from January to March. These winter indices were subsequently averaged at decadal time periods to adapt climate modes at the timescales of periodical VI values (Supplementary Fig. 3a).

Long-term monthly temperature and precipitation data were obtained from the 20CRV2c reanalysis data set^69^ provided by the NOAA/OAR/ESRL PSD, Boulder, Colorado, USA, from their web site at http://www.esrl.noaa.gov/psd/. Additionally, we used available monthly precipitation and temperature data recorded in the closest meteorological station to each forest site to assess winter precipitation, winter mean temperature, potential evapotranspiration (PET^70^), and the climatic winter water balance: i.e., precipitation-evapotranspiration (P-PET). We used winter climatic data (i.e., January–March) since winter water balance is critical for soil water recharges and thus trees are responsive to winter climate in such Mediterranean forest ecosystems^21^. Further information on geographical coordinates and periods of available data can be seen in Supplementary Table 3. Local climate data were all accessed in the Agencia Española de Meteorología (AEMET).

### Statistical analyses

First, we analyzed decadal forest unit VI as function of MTS, TD, wood Th, the calendar years, and the interaction NAO × AMO using general linear mixed-effect models. To account for the spatial arrangement of the data, forest units nested in forest landscapes were included as a random term and derived as variance random components in the model (i.e., landscapes/units). We selected the predictor variables in the fixed term of the model by applying the AIC corrected for small sample sizes (AICc) using a backward selection procedure. Firstly, we defined a full model according to our general VI hypothesis. Secondly, we created as many nested models as predictors in the fixed term and we evaluated their contribution as explicative variables using delta AICc (i.e., AICc of each model minus AICc of the full model). We included a predictor only if its elimination from the full model determines an increment of at least 4 units of AICc^71^. To test the interaction NAO × AMO, we compared the full model vs. a nested model with only the main effects of each variable (i.e., NAO + AMO). Model assumptions of homogeneity of variance and normality were checked using graphical analyses of residuals. We log-transformed the response variable to meet the normality assumptions. All the predictor variables were standardized before model fitting and multicollinearity was evaluated using the variance inflation factor (e.g., VIF). Goodness of fit was evaluated in a two-way procedure: graphical analysis of predicted vs. observed VI values, and, conditional *R* ^272^.

Second, we applied a structural equation model^36^ (SEM) to unravel the simultaneous indirect and direct effects of North Atlantic climatic modes (AMO and NAO), forest factors (MTS), climatic variability (winter water balance computed using winter precipitation and temperature in 20CRV2c reanalysis data set), and the calendar year as a surrogate of global change trends on decadal VI over the last century. We used SEM in an exploratory mode to obtain hypothesized causal relationships based on simultaneous mixed-effects models. On the one hand, we hypothesized that winter water balance (P-PET) can be modeled as function of North Atlantic climatic modes and the calendar year^35^. Analogously, VI can be expressed as a function of P-PET, the calendar year (i.e., carbon fertilization and enhanced water use efficiency due to rising atmospheric CO_2_ and N deposition^59,60^), and significant forest factors obtained in the previous mixed-effect model (i.e., MTS). The spatial arrangement of data imposes random terms with the form of forest landscapes and forest units nested in forest landscapes, respectively, for climatic and VI measurements. Independent claims (i.e., missing paths) and goodness of fit were assessed with a *χ* ^2^-test on the Fisher’s C statistic^73^. Models’ *R* ^2^ values derived from the variance of both fixed and random effects were calculated^72^. SEM analyses were conducted in R using the piecewiseSEM package^73^.

Third, we performed three separate mixed models to fit local climatic data at annual timescale resolution (i.e., winter total precipitation, mean temperature, and water balance obtained in five nearby meteorological stations) to interactive effects of NAO and AMO. We included the meteorological station as a random term affecting the intercept parameter and tested the fixed terms using a backward selection procedure. We used linear mixed models with normal error distribution when errors distribute normally and generalized mixed models with gamma distribution and log link function, otherwise. All models were conducted using the lme function of nlme package in the R environment^74^.

### Data availability

The data that support the findings of this study are available upon reasonable request from the corresponding author and from Mrs. Maria Bragado Jambrina (BraJamMa@jcyl.es) at the public institution Delegación Territorial de Medio Ambiente in Segovia, Spain.

## Electronic supplementary material

Supplementary information
