# Supplementary material for: Forest productivity in southwestern Europe is controlled by coupled North Atlantic and Atlantic Multidecadal Oscillations
Source: Nat Commun. 2017 Dec 20;8:2222. doi: 10.1038/s41467-017-02319-0 (PMC5738338; doi:10.1038/s41467-017-02319-0)
Supplement: Supplementary file 1 — Supplementary information [file 41467_2017_2319_MOESM1_ESM.pdf]

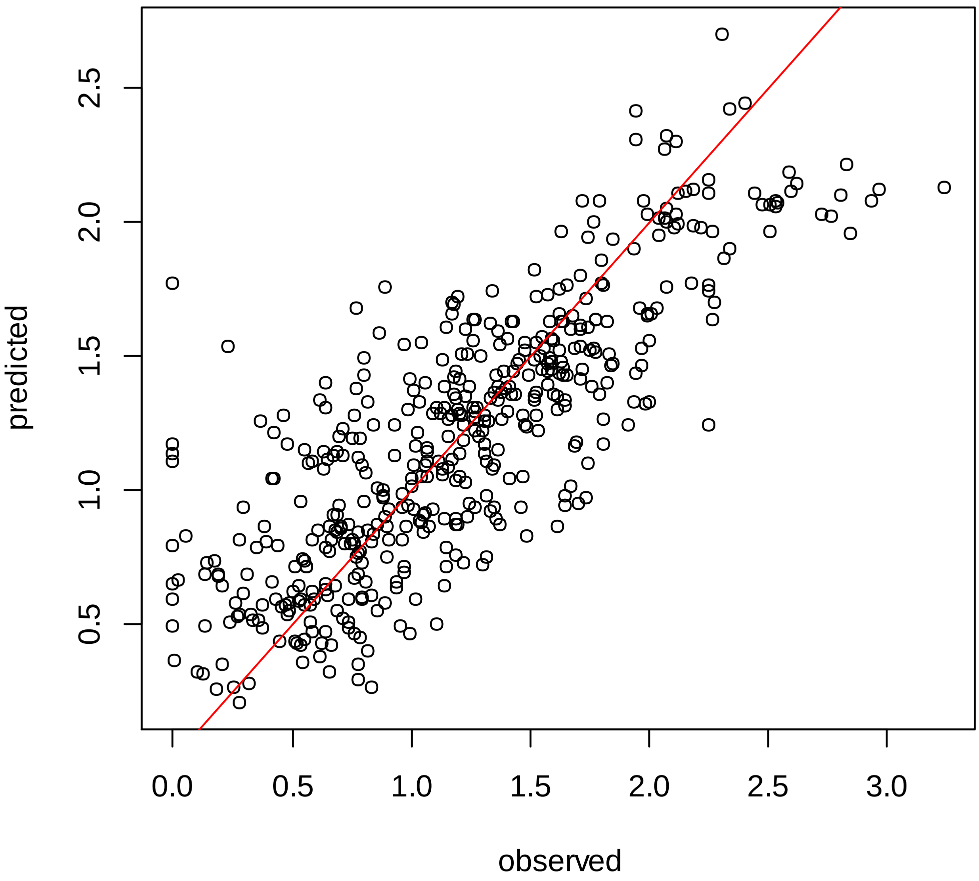

Supplementary Figure 1. Observed vs predicted values. Graphical evaluation of the goodness-of-fit using observed vs. predicted volume increment as function of NAO x AMO (Mixed-effects model)

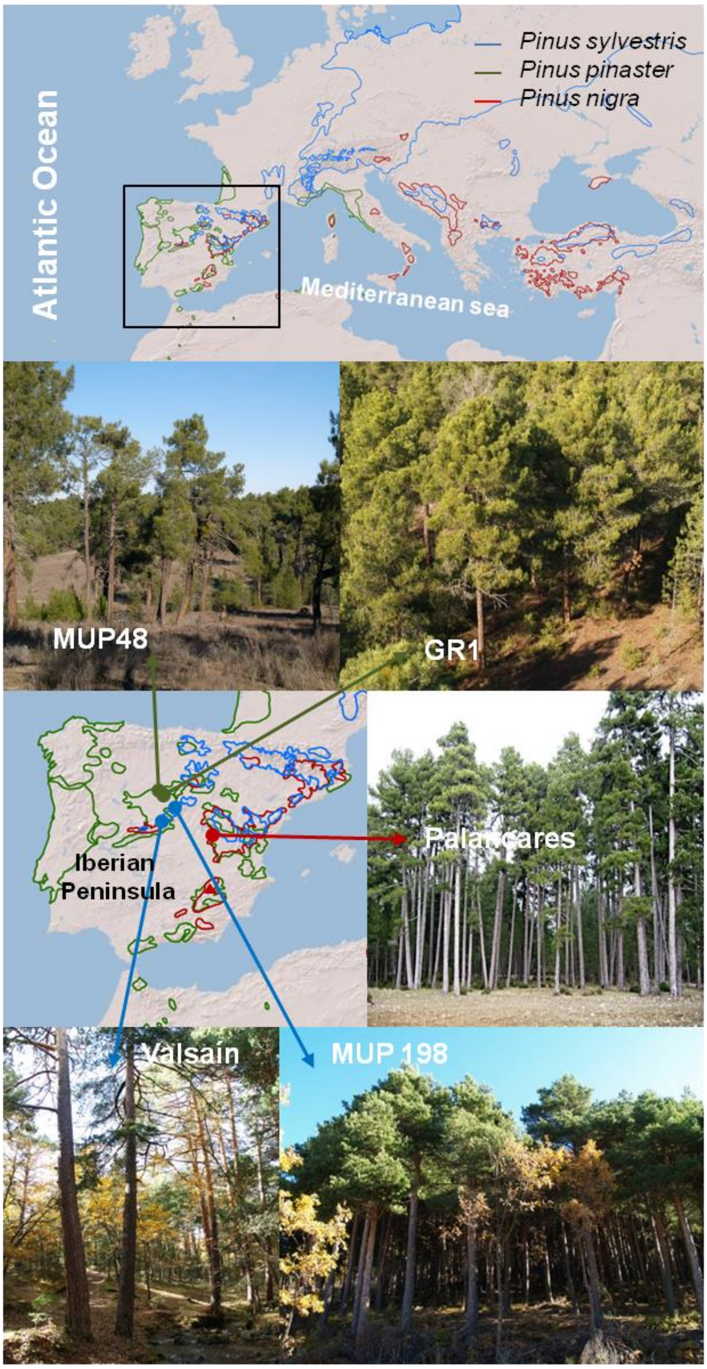

Supplementary figure 2. Pine forests distribution and appearance. Location of forest landscapes overimposed on species distribution ranges for the three species considered (*Pinus pinaster* – green, *Pinus nigra* – blue, *Pinus sylvestris* – red). Photos representative of each studied forest landscape are included. All images and maps were created by Dr. Jaime Madrigal-González and Dr. Manuel E. Lucas –Borja.

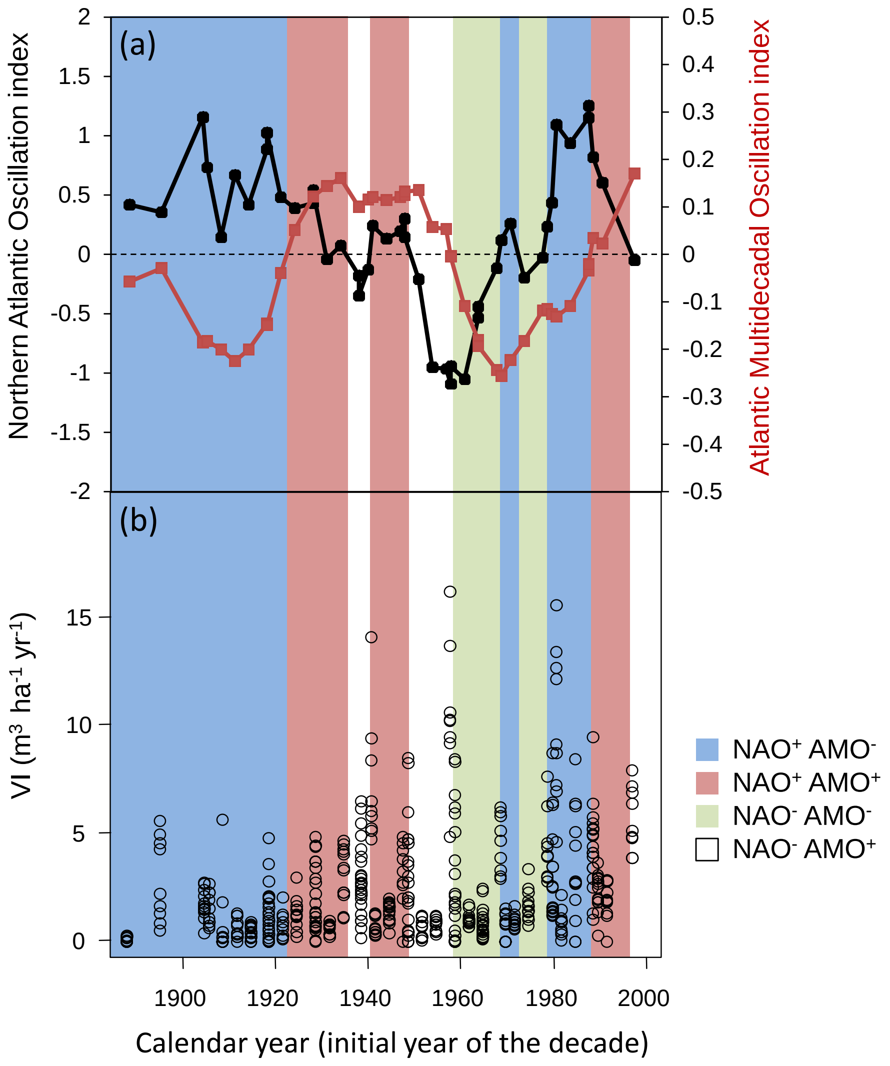

Supplementary figure 3. North Atlantic Oscillations and Volume increment data throughout time. (a) Averaged North Atlantic and Atlantic Multidecadal Oscilations (black and red lines, respectively) and (b) volume increment (VI) for each forest unit in periods between consecutive inventories. In the x-axis the calendar year is indicative of the initial year of each decadal period (see Table 3 for more details). Vertical colored bars in the bottom of the figure represent one out of four potential combinations between NAO and AMO in the temporal series from 1889 to 2010.

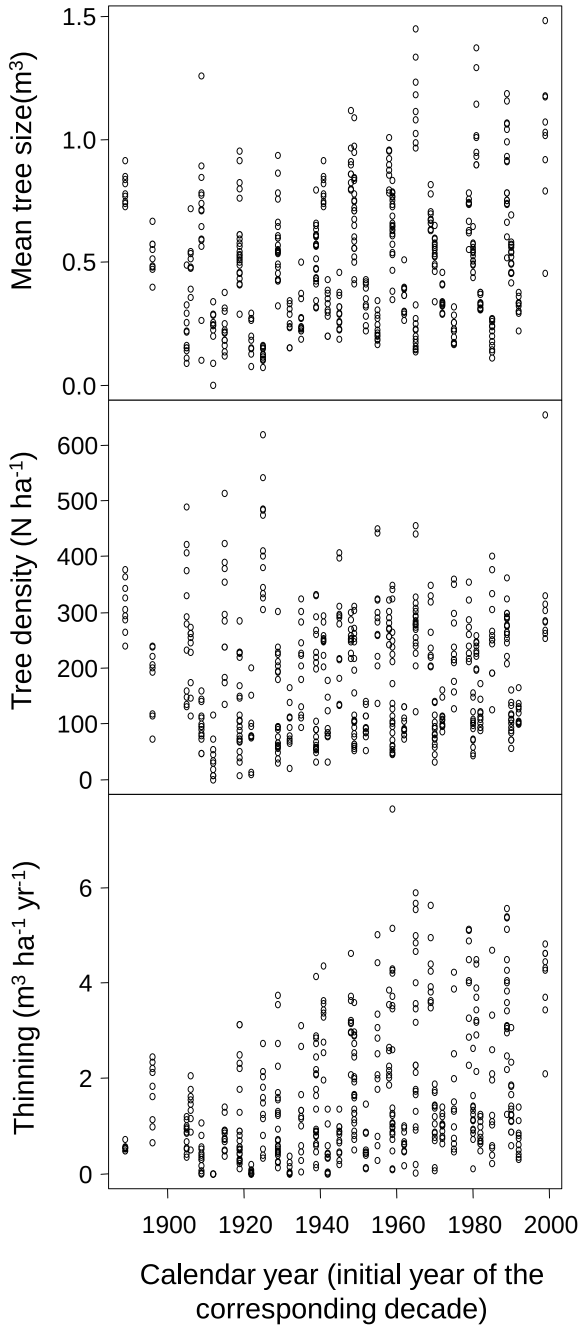

Supplementary figure 4. Forest data throughout time. Evolution of the confounding information associated with forest stocks (mean tree size and tree density) and management (thinning) throughout the 20th century.

Tables

Supplementary table 1. Variable selection using a backward procedure based on the Akaike Information Criterion corrected for small sample sizes (AICc). ∆AICc is the AIC difference between the reference model (full model) and models without the fixed effect indicated in the action. The column 'Selection' indicates supported (s) and non-supported (is) variables in the backward selection process based on the ∆AICc criterion (i.e. ∆AICc > 4 to select the variable**)**. In a second round (last two lines of the table) the final supported model (in bold letters) was compared to a null model without predictor variables which is indicative of an absence of environmental effects. Selection column: is – insufficient support, s – supported. Model terms legend: SP – species identity; MTS– mean tree size; Th – thinning (harvested wood volume); TD – tree density; yr – calendar year (last-century global change trends); AMO – Atlantic Multidecadal Oscillation; NAO – Northern Atlantic Oscillation.

**Models**	**Action**	**AICc**	**∆AICc**	**Selection**
SP + MTS + Th + TD + yr + (AMO × NAO)	Reference model	525.1114	0	-
MTS + Th + TD + yr + (AMO × NAO)	SP eliminated	525.9129	0.8014312	is
SP + Th + TD + yr + (AMO × NAO)	MTS eliminated	533.0096	7.898131	s
SP + MTS + TD + yr + (AMO × NAO)	Th eliminated	522.8714	-2.239993	is
SP + MTS + Th + yr + (AMO × NAO)	TD eliminated	518.3502	-6.761196	is
SP + MTS + Th + TD + (AMO × NAO)	yr eliminated	578.5741	53.46271	s
SP + MTS + Th + TD + yr + AMO + NAO	AMO × NAO eliminated	531.6724	6.560991	s
**MTS + yr + (AMO × NAO)**	**Best model**	**517.333**	**0**	**s**
Null Model (intercept only)	All variables eliminated	597.7559	72.64448	

Supplementary table 2. Parameter estimates and standard errors for the best model of forest productivity. MTS – mean tree size; yr – calendar year; AMO – Atlantic Multidecadal Oscillation; NAO – Northern Atlantic Oscillation. The Variance Inflation Factor (VIF) is shown as a multicollinearity test.

	**Estimate**	**Std. Error**	**VIF**
(Intercept)	1.2332359	0.30015804	-
MTS	-0.164204	0.04122049	1.09024
Yr	0.220362	0.01954902	1.19839
AMO	0.0728435	0.01813594	1.04884
NAO	-0.007214	0.01845294	1.10335
AMO x NAO	-0.103467	0.02699581	1.17563

Supplementary table 3. Summarized information for the Meteorological Stations used to calculate local climatic conditions. Time periods of available data represent periods without gaps in any of the variables considered (i.e. temperature or precipitation).

**Village/Town**	**Coord X**	**Coord Y**	**Altitude**	**Data**	**Period**	**Forest**
Valladolid	-4.77E	41.65N	727	Prec/Temp	1951-2017	MUP48, MUPGR1
Zamarramala	-4.13E	40.96N	1000	Prec/Temp	1950-1995	MUP48, MUPGR1
Puerto Navacerrada	-4.00E	40.78N	1890	Prec/Temp	1946-2017	MUP_Valsain
Rascafría	-3.87E	40.90N	1139	Prec	1945-1997	MUP198
Cuenca	-1.54E	39.34N	945	Prec/Temp	1956-2017	Palancares

Supplementary table 4. Parameter estimates for generalized linear regression models fitted to (a) winter precipitation (Prec, R^2 = 0.42), (b) winter mean temperature (MT, R^2

= 0.87), and (c) winter water balance (P-PET, R^2 = 0.59) as function of NAO x AMO, calendar year and the meteorological station (factor with five categories).

(a)	Estimate	Std. Error	t value	significance
(Intercept)	0.007677	0.000462	16.594	***
Station (Nav)	-0.004656	0.000481	-9.680	***
Station (Val)	0.001654	0.000690	2.396	*
Station (Seg)	0.000216	0.000687	0.315	ns
AMO	0.000406	0.000147	2.759	**
NAO	0.000922	0.000144	6.414	***
AMO x NAO	0.000355	0.000124	2.862	**
Year	0.000138	0.000141	0.973	ns

(b)	Estimate	Std. Error	t value	significance
(Intercept)	5.94464	0.14107	42.138	***
Station (Nav)	-5.74410	0.19105	-30.066	***
Station (Val)	0.01716	0.18962	0.090	ns
Station (Seg)	0.20977	0.21017	0.998	ns
AMO	0.35669	0.07181	4.967	***
NAO	0.14791	0.07227	2.047	*
AMO x NAO	0.10918	0.06552	1.666	ns
Year	0.32916	0.07212	4.564	***

(c)	Estimate	Std. Error	t value	significance

(Intercept)	0.007677	0.000462	16.594	***
Station (Nav)	-0.004656	0.000481	-9.680	***
Station (Val)	0.001654	0.000690	2.396	*
Station (Seg)	0.000216	0.000687	0.315	ns
AMO	0.000406	0.000147	2.759	**
NAO	0.000922	0.000144	6.414	***
AMO x NAO	0.000355	0.000124	2.862	**
Year	0.000138	0.000141	0.973	ns

Supplementary table 5. Summary of geographical, environmental, and management information in the studied forest landscapes. Forest units are subareas within forests that can be considered pseudo-replicates of each forest landscape.

**Forest landscape**	**MUP48**	**MUPGR1**	**MUP198**	**MUP_Valsaín**	**Palancares**
X cords.	4º11'44"W	4º05'48"W	3º50'00"W	4º02'01"W	1º59'10"W
Y cords.	41º18'32"N	41º15'18"N	41º00'52"N	40º02'01"N	40º01'50"N
Location	Cuéllar	Aguilafuente	Navafría	Valsaín	Palancares
Altitude (m.a.s.l.)	800-850	850-970	1300-1900	1200-1900	1100-1300
Forest area (ha)	6828	6761	3831	10672*	4910
No. of forest units	10	14	9	8	12
Rotation period (No. years)	100	100	120	120	120
Starting year	1912	1896	1895	1899	1905
No. inventories	9	9	10	8	9
Lithology	Sands	Sands	Granites	Granites	Limestone
Annual precipitation (mm)	430	450	524-1000	885-1170	595
Mean annual temperature (ºC)	11.9	11.7	6.9-10.1	6.9-10.3	9.6
Species	*P. pinaster*	*P. pinaster*	*P. sylvestris*	*P. sylvestris*	*P. nigra*

* Subdivided in turn into two large forests: Monte Pinar and Monte Matas (7622 ha and 3046 ha respectively). We used units of the Monte Pinar data since is the oldest record in this large forest landscape.
